# Characterization of Novel HIV-1 Intersubtype CRF01_AE/C and A1/C Recombinants from India

**DOI:** 10.1128/genomeA.00849-15

**Published:** 2015-08-20

**Authors:** Sudhanshu Shekhar Pandey, Madhuri Thakar, Ramesh Paranjape

**Affiliations:** Department of Immunology, National AIDS Research Institute (ICMR), Pune, India

## Abstract

We report here three novel HIV-1 intersubtype recombinants from India. One among those is a recombinant between subtype C and CRF01_AE and another two between A1 and C. A recombinant virus with CRF01_AE is reported for the first time from India.

## GENOME ANNOUNCEMENT

Human immunodeficiency virus strains have shown extraordinary genetic diversity, as evidenced by 9 subtypes, >70 circulating recombinant forms (CRFs), and innumerable unique recombinant forms. Of these, subtype C is most prevalent in India, while subtypes A1, B, CRF01_AE, and CRF02_AG have occasionally been reported. Apart from these, A1/C (1, 2) and B/C (3) intersubtype recombinants have also occasionally been reported. Here, we report for the first time in India a unique HIV-1 recombinant between subtype C and CRF01_AE and two novel recombinants between subtype A1 and C. Unique recombinant forms (URFs) between CRF01_AE and subtype C have been reported from Myanmar, China, and Nepal (Los Alamos HIV Database).

The recombinant strain NARI-FLS_VB5 was amplified using cocultured proviral DNA using a methodology described previously (4) and sequenced directly from the PCR product using the ABI Prism 3730XL DNA analyzer (Applied Biosystems). The near-full-length genome (NLFG) sequence was initially analyzed for recombination using RIP and for recombination breakpoints using jpHMM at Gobics and SimPlot. Breakpoints were further confirmed using phylogenetic analysis.

Strain NARI-FLS_VB-5 showed an insertion of CRF01_AE at three sites, positions 5711 ± 9 to 5901 ± 8, 6025 ± 13 to 8380 ± 7, and 8868 ± 6 to 8978 ± 6 into the backbone of subtype C, whereas at positions 8380 ± 7 to 8593 ± 7, jpHMM showed uncertainty between subtype C and CRF01_AE. A bootscan analysis of NARI-FLS_VB-5 showed similar breakpoints to those of jpHMM, with two more small insertions of CRF01_AE at positions 2871 to 3050 and 4878 to 5013. Combining the results obtained with RIP (5), jpHMM (6), and SimPlot (1) suggests insertions of CRF 01_AE at five positions in the backbone of subtype C.

Two of the strains, NARI-FLS_VB27 and NARI-FLS_IVC3, have been cloned and sequenced using a methodology described previously (4). NARI-FLS_VB27 and NARI-FLS_IVC3-1 showed recombination of subtype A1 into the backbone of subtype C. Both clones of NARI-FLS_IVC3-1 showed an insertion of subtype A1 into the backbone of subtype C in *pol* (positions 3508 ± 17 to 4033 ± 35) and *nef*-*LTR* (positions 8578 ± 9 to 9632). Of the 11 clones generated for NARI-FLS_VB27, six clones showed two small insertions of subtype A1 into the backbone of subtype C int-*vif* (positions 4826 ± 84 to 5162 ± 10 and 5368 ± 10 to 5570 ± 26); one clone showed slightly different breakpoints (positions 4826 ± 84 to 5202 ± 20 and 5455 ± 24 to 5570 ± 26), and four clones showed an insertion of subtype A1 at positions 4826 ± 84 to 5162 ± 10 and uncertainty between subtype A1 and C at positions 5436 ± 5 to 5583 ± 12. Phylogenetic analysis using a recombinant region from positions 5368 to 5583 formed a monophyletic cluster with bootstrap support of 100. The phylogenetic tree suggests that the uncertainty region may be a result of the evolution of the strain after the introduction into the subject. SimPlot analysis also showed breakpoints in concordance with jpHMM (6) for both strains. All the breakpoints used in the study were relative to the HXB2 genome.

A comparison of the URFs characterized in this study with the earlier reported URF 01C and A1C recombinant forms suggest that all three characterized URFs are novel recombinants. The detection of an 01C recombinant also points to the fact that HIV viruses belonging to multiple subtypes are circulating and giving rise to new URFs. This clearly indicates that the HIV epidemic in India is still evolving and needs close monitoring.

### Nucleotide sequence accession numbers.

The near-full-genome shotgun sequences are available in GenBank under the accession numbers KT175202 to KT175215.
